# Case of Poisoning with Arsenic

**Published:** 1850-10

**Authors:** A. G. Greaves

**Affiliations:** Surgeon to the Derby Dispensary


### PATHOLOGY AND PRACTICE OF MEDICINE.

*Case of Poisoning with Arsenic. Death in thirty-six hours—Influence of Arsenic on Digestion—Remission of Symptoms.* By A. G. Greaves, Surgeon to the Derby Dispensary.—On the 26th of February, 1847, Mrs. Ann W——, aged 24, in the ninth month of pregnancy, was taken ill at half past 9 A. M., with sickness and vomiting, having an hour previously partaken of a breakfast of tea and bread and butter. The vomiting continued at intervals during the day, the matter thrown up consisting principally of bile. No medical assistance was called in, deceased saying that she thought her illness was owing to her pregnancy.

27th.—Mrs. W. vomited once at 9 A. M., and then felt very much better. She cleaned her house thoroughly during the morning, took some gruel at half-past 12, and at 1 P. M. left home, as she said for a walk. She returned at 2 P. M. and stated that she had been obliged to call at the house of a friend about two hundred yards from her home, having again been sick. At half-past two she again threw up a quantity of what, from the description given, I supposed to be bile. At half-past 4 I was called in, and found her seriously ill, complaining of violent pain in the stomach and abdomen, with diarrhœa and tenesmus, and great difficulty of breathing. She sank rapidly, and died at a quarter past 7 P. M.

I ascertained that deceased had felt the movement of the child a very short time before her death, and I therefore urged the friends to allow me at once to open the uterus and remove the fœtus; but they objected.

*Post-mortem* 22 *hours after death.*

*Head.*—Brain and the membranes perfectly healthy.

*Chest.*—Lungs contained a considerable quantity of dark blood. The heart was very flaccid, and both ventricles were filled with fluid blood of a very dark character.

*Stomach* contained about a pint and a half of bloody fluid, on pouring off which I found a very large quantity of exceedingly viscid albuminous secretion containing numerous patches of a white powder, which analysis proved to be arsenic. The mucous membrane was much inflamed.

The duodenum was also inflamed, and contained one or two patches of the same white powder. In the caput coli was found a considerable quantity of *undigested* soup, containing several patches of undissolved arsenic, precisely like those in the stomach.

The bladder was empty and healthy. The uterus contained a fine healthy child, but there was no evidence of labor being near. The membranes were entire, and everything in a normal state. A careful analysis by myself and my friend, Mr. Bernays, shows most unequivocally the presence of a large portion of arsenious acid in the contents of the stomach, but by whom administered there was no evidence to show, beyond a remark of the deceased, "that it would be a shocking thing if she and her husband should be found dead in bed." This led to a suspicion that it was an act of suicide.

We have unfortunately, no satisfactory data to form a conclusion as to when the *fatal* dose of arsenic was taken. There can be no doubt that some of the poison had been administered on the 26th, thirty-six hours before death, but whether that was the dose of which she died is uncertain. It is interesting to remark how large a portion of arsenic remained in the stomach, notwithstanding the continued vomitings the deceased had for five hours before death.

There are some circumstances connected with this case which are of interest in a pathological view. It appears that the deceased partook of of a quantity of soup about *seven* hours before her death; and yet, although this long period had elapsed, it was found in the caput coli in an undigested state, and mixed with a quantity of arsenic. But for this fact being well known, it might have been inferred that deceased had taken a large dose of arsenic in soup three or four hours before death. This appears to show that the action of arsenic materially interferes with the process of healthy digestion.

Another circumstance worthy of notice is, that there was so complete a remission of the symptoms after the poison had been swallowed, that the deceased was able to occupy herself in her usual avocations, and to go for a walk.—*Medical Gazette.*

